# Molecular diversity of extended-spectrum β-lactamases and carbapenemases, and antimicrobial resistance

**DOI:** 10.1186/s40560-020-0429-6

**Published:** 2020-01-28

**Authors:** Teiji Sawa, Kunihiko Kooguchi, Kiyoshi Moriyama

**Affiliations:** 1 Department of Anesthesiology, School of Medicine, Kyoto Prefectural University of Medicine, 465 Kajii-cho, Kamigyo, Kyoto, 602-8566 Japan; 2 Department of Intensive Care, Kyoto City Hospital, 1-2 Higashitakada-cho, Mibu, Nakagyo, Kyoto, 604-8845 Japan; 3 Department of Anesthesiology, School of Medicine, Kyorin University, 6-20-2 Shinkawa, Mitaka, Tokyo 181-8611 Japan

**Keywords:** β-Lactam, β-Lactamase, Carbapenemase, Classification, Multidrug resistance

## Abstract

Along with the recent spread of multidrug-resistant bacteria, outbreaks of extended-spectrum β-lactamase (ESBL) and carbapenemase-producing bacteria present a serious challenge to clinicians. β-lactam antibiotics are the most frequently used antibacterial agents and ESBLs, and carbapenemases confer resistance not only to carbapenem antibiotics but also to penicillin and cephem antibiotics. The mechanism of β-lactam resistance involves an efflux pump, reduced permeability, altered transpeptidases, and inactivation by β-lactamases. Horizontal gene transfer is the most common mechanism associated with the spread of extended-spectrum β-lactam- and carbapenem resistance among pathogenic bacterial species. Along with the increase in antimicrobial resistance, many different types of ESBLs and carbapenemases have emerged with different enzymatic characteristics. For example, carbapenemases are represented across classes A to D of the Ambler classification system. Because bacteria harboring different types of ESBLs and carbapenemases require specific therapeutic strategies, it is essential for clinicians to understand the characteristics of infecting pathogens. In this review, we summarize the current knowledge on carbapenem resistance by ESBLs and carbapenemases, such as class A carbapenemases, class C extended-spectrum AmpC (ESAC), carbapenem-hydrolyzing class D β-lactamases (CHDLs), and class B metallo-β-lactamases, with the aim of aiding critical care clinicians in their therapeutic decision making.

## Background

Among the recent spread of multidrug-resistant bacteria, outbreaks of extended-spectrum β-lactam- and carbapenem-resistant bacteria are a serious problem not only making treatment difficult but also worsening the prognosis of infected patients [1]. β-lactam antibiotics are the most frequently used antibacterial agents, and extended-spectrum β-lactams and carbapenems have been developed as specific drugs to treat bacterial species resistant to penicillins and cephems [2]. Bacterial resistance to carbapenems incorporates not only carbapenem antibiotics but also resistance to penicillin and cephem antibiotics. Therefore, resistance to carbapenems presents a significant threat to patients who are immunocompromised and are therefore susceptible to infections caused by multidrug-resistant bacteria all over the world [3]. The mechanism of resistance to extended-spectrum β-lactams and carbapenems involves an efflux pump, reduced permeability, altered transpeptidases, and inactivation by β-lactamases. Horizontal gene transfer is the most common mechanism associated with the spread of antimicrobial resistance across pathogenic bacterial species, such as carbapenemase-producing *Enterobacteriaceae* (CPE) [4, 5]. In various bacterial species, novel extended-spectrum β-lactamases (ESBLs) or carbapenemases with different structures or characteristic features are reported each year.

Various ESBLs and carbapenemases have been reported in the *Enterobacteriaceae* including *Enterobacter*, *Klebsiella*, *Escherichia coli* [6, 7], and other opportunistic species such as *Serratia*, *Acinetobacter*, and *Pseudomonas* [8]. In addition, the genetic elements by which drug-resistant genes horizontally move across bacterial species have been studied among these bacterial species. As one typical example, *Pseudomonas aeruginosa*, one of the major causative agents of infection in immunocompromised individuals, displays resistance to various antibacterial agents. Its resistance mechanism is, in part, derived from the organism’s natural resistance, but is also acquired through horizontal gene transfer and/or mutations within its DNA. *P. aeruginosa* is naturally resistant to β-lactam antibiotics, such as penicillin and cephem, and aminoglycoside antibiotics. Since the 1990s, *P. aeruginosa* strains have emerged that have acquired resistance to broad-spectrum penicillins, third-generation cephems, carbapenems, anti-*P. aeruginosa* aminoglycosides, and new quinolones [9, 10]. Among these multidrug-resistant *P. aeruginosa* (MDRP), one of the major clinical concerns is the spread of *P. aeruginosa* strains that harbor a carbapenemase because β-lactam antibiotics including carbapenems are the most frequently used antibacterial agents. An anti-methicillin-resistant *Staphylococcus aureus* (MRSA) drug, albekacin sulfate, a monobactam aztreonam, and polypeptide colistin appear to be effective against MDRP, but the emergence of resistant strains has also been reported including extensively drug-resistant *P. aeruginosa* (XDRP) and pandrug-resistant *P. aeruginosa* (PDRP) [10, 11].

To date, many different types of ESBLs and carbapenemases have emerged with different enzymatic characteristics [1]. Because the specific therapeutic strategy is dependent on the type of ESBL and carbapenemase, it is vital for clinicians to understand the characteristics of ESBLs and carbapenemases [12]; however, in practice, the complex biology associated with ESBLs and carbapenemases presents significant challenges in the effective control of infections. In this review, we summarize antimicrobial resistance by ESBLs and carbapenemases, with the aim of collating the current knowledge in this field to aid therapeutic decision making by critical care clinicians.

## β-Lactam antibiotics, penicillin-binding protein (PBP), and β-lactamase

**Fig. 1:**
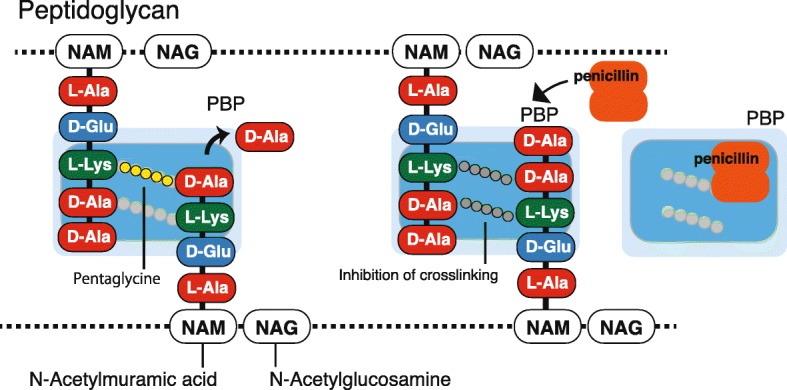
The antimicrobial action of β-lactamase against the peptidoglycan structure of the bacterial cell membrane. Peptidoglycan possesses a basic structural unit in which two amino sugars of *N*-acetylglucosamine (NAG) and *N*-acetylmuramic acid (NAM) alternate, and a longitudinal peptide chain linked to NAM forms a pillar. This peptide chain is a pentapeptide consisted of alternating d- and l-forms of l-alanine, γ-d-glutamic acid, l-lysine, d-alanine, and d-alanine that forms a bacterial enzyme known as penicillin-binding protein (PBP). PBP recognizes alanyl-alanine, which is an alanine dimer formed by d-alanine-d-alanine present at the end of the pentapeptide, and exerts the enzymatic action of cross-linking. Since penicillin is structurally similar to alanyl-alanine at the terminal region of the pillar structure, PBP captures penicillin, thereby inhibiting the cross-linking reaction

We will begin by reiterating the mechanism of action of β-lactam antibiotics. The main constituent of the cell wall formed in the outer membrane layer of eubacteria is peptidoglycan, which is a macromolecular structure consisting of peptide and sugar (Fig. 1). This peptidoglycan structure confers resistance to osmotic pressure and retains cell morphology and strength. It is also the target of β-lactam drugs. By inhibiting the formation of this structure, β-lactams suppress bacterial cell division (bacteriostatic action) or induce bacterial rupture against osmotic pressure (bactericidal activity). Peptidoglycan possesses a basic structural unit in which two amino sugars of *N*-acetylglucosamine (NAG) and *N*-acetylmuramic acid (NAM) alternate, and a longitudinal peptide chain linked to NAM forms a pillar. This peptide chain is a pentapeptide consisting of alternating d- and l-forms of l-alanine, γ-d-glutamic acid, l-lysine, d-alanine, and d-alanine residues. At the time of cross-linking, d-alanine at the carboxyl-terminal of pentapeptide is first eliminated by hydrolysis. Next, the fourth d-alanine carboxyl group and the third diaminopimelic acid, which is a ε-carboxyl derivative of lysine of the same columnar structure as the neighboring molecule, are combined with a triglycine peptide structure for horizontal reinforcement of the chain. These reactions are mediated by the bacterial enzyme known as PBP.

**Fig. 2:**
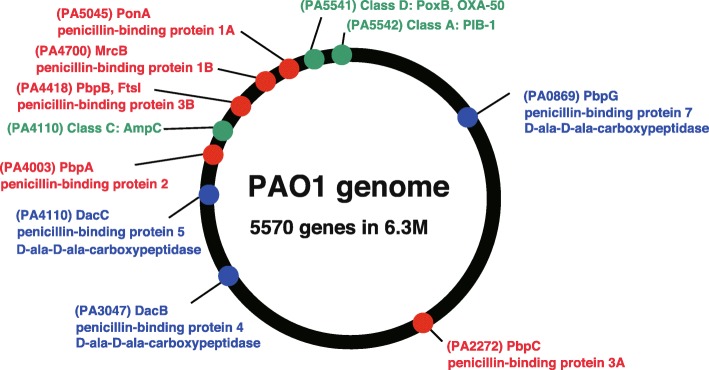
The genes encoding penicillin-binding proteins in *P. aeruginosa* PAO1. Eight types of PBP genes and three chromosomal β-lactamase genes for PIB-1 (class A), AmpC (class C), and PoxB (class D) exist in the genome of *P. aeruginosa* reference strain PAO1 [15]

PBP recognizes alanyl-alanine, which is an alanine dimer formed by d-alanine-d-alanine present at the end of the pentapeptide, and its enzymatic activity mediates cross-linking [13, 14]. A cross-linking reaction is induced by the formation of a covalent bond between the serine residue in the active center of PBP and the carboxyl group produced by hydrolytic cleavage of d-alanine. Thereby, PBP displays adenyl-alanine endopeptidase activity. Since penicillin has a structure similar to that of alanyl-alanine at the terminal part of this pillar structure, PBP cannot distinguish between these structures and binds to both, leading to inhibition of the cross-linking reaction. Seven types of PBP genes exist in the *E. coli* genome, and eight types of PBP genes exist in the genome of *P. aeruginosa* reference strain PAO1 (Fig. 2) [15]. Among them, five types of genes (PBP1A, PBP1B, PBP2, PBP3A, and PBP3B) encode high molecular weight PBPs (HMM-PBPs, molecular weight 60,000 to 90,000) that display both transglycosylase and transpeptidase activities and play a role in cell elongation and partition formation. In cell division and morphogenesis, PBP1A and PBP1B are thought to be involved in growth and elongation, PBP2 in formation of a gonococcal form, and PBP3 in partition formation during division.

The remaining three types are low molecular mass BPBs (LMM-PBPs; molecular weight of 40,000–50,000) that display d-alanine carboxypeptidase activity and are widely resistant to various β-lactams. The roles of LMM-PBPs are still under research. Both the HMM-PBP transpeptidase activity and the LMM-PBP alanine carboxypeptidase activity utilize a serine residue as an enzymatic activity center, and the β-lactam antibacterial agent exhibits enzyme inhibitory activity by binding to this serine residue, which is a common feature of class A, C, and D β-lactamases (which is discussed again later). As one mechanism of the development of resistant bacteria, the substrate specificity of PBP may be changed to reduce the ability to link to a β-lactam-based antibacterial agent. For example, MRSA produces PBP2′, which possesses a mutation in PBP, that has low affinity for β-lactams. Another example is the mechanism of resistance to the glycopeptide class of antibacterial agents, such as vancomycin. In this mechanism, *Enterococci* with the *vanA* gene cluster derived from transposon Tn1546 expresses a group of enzymes that exchange the peptidoglycan peptide longitudinal chain from d-alanine-d-alanine to d-alanine-d-serine and inhibit binding to vancomycin.

**Fig. 3:**
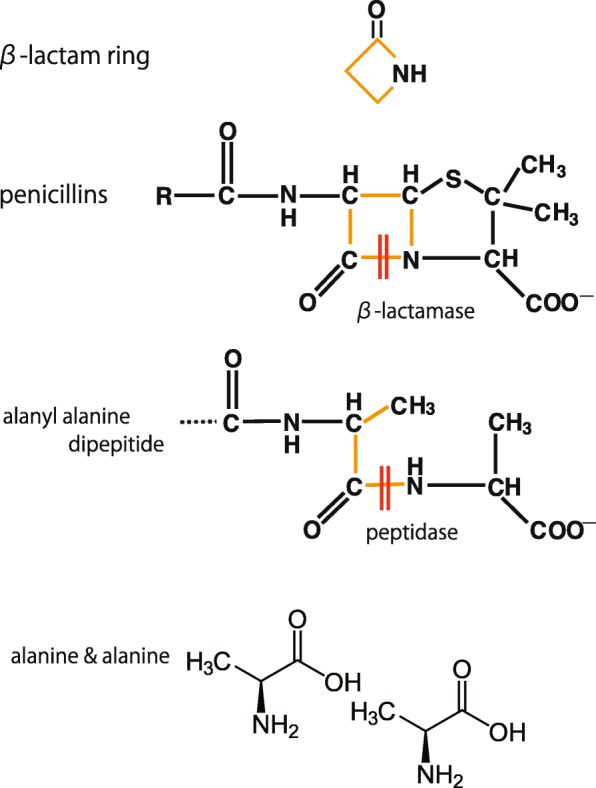
The enzymatic action of a β-lactamase to penicillins. The β-lactam ring is the basic structure of β-lactam antibiotics. β-lactamase inhibits the antibacterial action by dissociating the –CO–NH structure of the β-lactam ring. The –CO–NH structure of the β-lactam ring is similar to the –CO–NH peptide in alanyl-alanine, from which the peptidoglycan cross-links are formed. Thus, β-lactams mimic alanyl-alanine of the peptidoglycan pillar structure. Thus, β-lactamase is a protease (peptidase) that dissociates the –CO–NH structure of β-lactams

Next, we will reiterate the mechanism of action of β-lactamase. β-lactamase inhibits antibacterial activity by dissociating the –CO–NH structure of the β-lactam ring, which is part of the basic structure of β-lactams (Fig. 3). The –CO–NH structure of the β-lactam ring comprises the –CO–NH peptide in the alanyl-alanine dimer from which the peptidoglycan cross-links are formed. Thus, β-lactams mimic the alanyl-alanine of the peptidoglycan pillar structure and this is the binding region. Thus, β-lactamase is a protease (peptidase) that dissociates peptide bonds. PBP is bound by the original substrate alanyl-alanine and penicillin, which works as an inhibitor since it shares a similar structure with alanyl-alanine. These findings indicate that PBP and β-lactamase share similar structures because they exert peptidase activity, implying that β-lactamase may be evolutionarily derived from PBP.

**Fig. 4:**
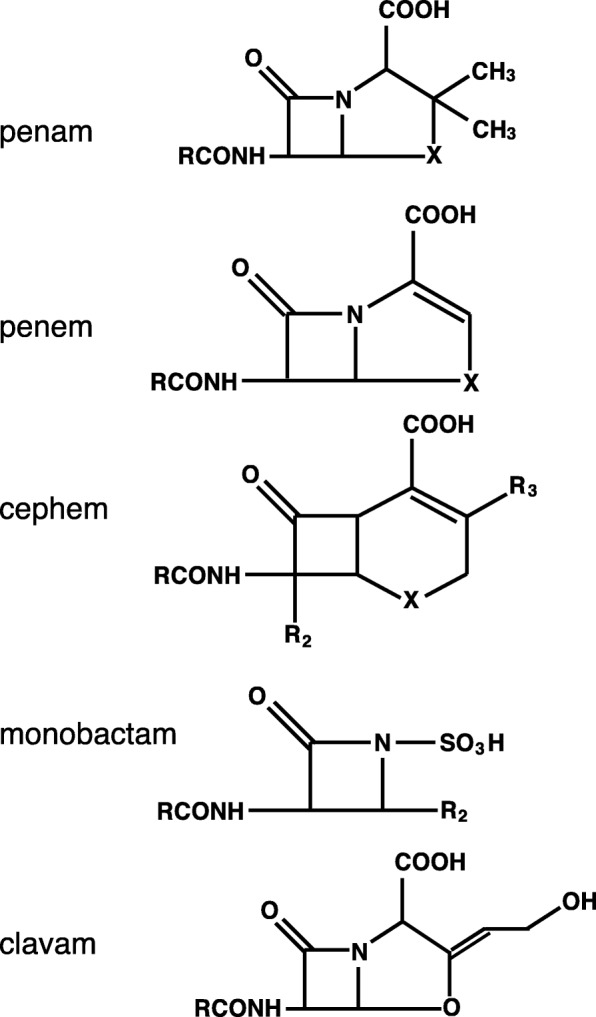
The chemical structure of β-lactam antimicrobials. Clinically used β-lactams can be classified into five basic structures capable of exerting different antimicrobial activities

In fact, the enzyme active site motifs S-X-X-K, S-D-N, and K-S/T-G in the primary sequence of class A β-lactamases, also exist in PBP (Additional file 1: Figure S1). This indicates that the β-lactamases and the peptidoglycan cross-linking enzyme, PBP, co-evolved. At present, clinically used β-lactams can be classified into five basic structures capable of exerting different antimicrobial activities (Fig. 4). Many of them originate from natural antibacterial substances produced by actinomycetes or fungi. Thus, actinomycetes themselves are resistant to their own antimicrobial agents. This indicates that both antibacterial substances and agents that counter such substances co-evolved in actinomycetes.

## Classification of β-lactamases and *P. aeruginosa* drug resistance

**Fig. 5:**
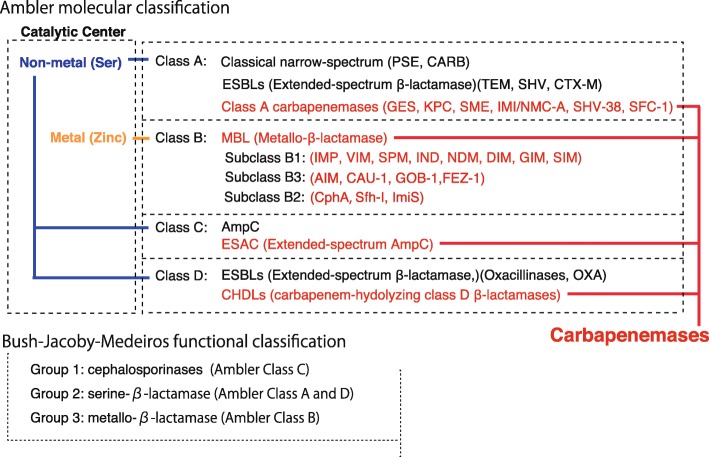
The classification of β-lactamases. Molecular structure classification using the Ambler method [16] and functional classification using the Bush-Jacobi-Medeiros method [17, 18] have both been employed. In the Ambler classification, β-lactamases are grouped into four classes A, B, C, and D by motifs composed of primary sequences constituting the protein molecules. β-lactamases of classes A, C, and D use a serine at the enzyme active center, whereas β-lactamases of class B use metal zinc ions. In functional classification using the Bush-Jacobi-Medeiros method, β-lactamases are classified into groups 1 to 3 depending on the degradation of β-lactam substrates and the effect of the inhibitor

To date, β-lactamases have been classified based on the molecular structure classification of Ambler [16] and the functional classification of Bush-Jacobi-Medeiros (Fig. 5) [17, 18]. In the Ambler classification, β-lactamases are grouped into four class A, B, C, and D according to motifs composed of primary sequences constituting the protein molecules. β-lactamases of classes A, C, and D use a serine as an enzyme active center, whereas β-lactamases of class B use the metal zinc. In the functional classification of Bush-Jacobi-Medeiros, β-lactamases are classified into groups 1 to 3 depending on the degradation of β-lactam substrates and the effects of inhibitors. Group 1 comprises cephalosporinases that are classified as class C based on molecular structural classification, and the gene involved was originally chromosomal. Group 2 comprises β-lactamases other than those in group 1 having serine in the active center and includes both classes A and D according to molecular structural classification. Group 3 comprises metallo-β-lactamases (MBLs) and corresponds to class B of the molecular structural classification. Within the genome of *P. aeruginosa* PAO1, a standard strain of *P. aeruginosa* and the first strain subjected to whole-genome sequence analysis, the class A PIB-1 (PA5542), class C AmpC (PA 4410), and class D PoxB (OXA-50, PA5541) β-lactamases are encoded (Fig. 2). Thus, *P. aeruginosa* PAO1 is equipped with three different types of β-lactamases, only lacking a class B β-lactamase [15]. These chromosome-encoded intrinsic β-lactamase genes of *P. aeruginosa* are partially responsible for its natural resistance to penicillins and cephalosporins.

## ESBLs and carbapenemases

ESBLs degrade third-generation cephem systems (such as cefotaxime and ceftazidime) [17] and are characterized by whether they are inhibited by β-lactamase inhibitors, such as clavulanic acid, sulbactam, and tazobactam [17]. The appearance of ESBLs in organisms such as *Klebsiella* and *E. coli* indicates that β-lactamase-producing genes, such as TEM-type and SHV-type genes, encoded on a plasmid (R plasmid), have broadened the range of target drugs by genetic mutation [12, 19]. Because the resistance genes are derived from the plasmid, they are easily transmitted among different bacterial species belonging to the same genera, such as the *Enterobacteriaceae*. The repeated mutation of ESBLs has contributed to the emergence of new β-lactamases, namely carbapenemases, which hydrolyze carbapenem antibiotics. In all classes A to D, based on molecular structural classification, β-lactamases that degrade carbapenems have been identified and are discussed below.

### Class A penicillinases, ESBLs, and carbapenemases

Class A penicillinases, such as PC1, which belong to the Bush-Jacoby functional subgroup 2a, hydrolyze a relatively limited spectrum of penicillins and are predominantly found in Gram-positive cocci. As class A ESBLs that degrade the early cephalosporins and belong to the Bush-Jacoby functional subgroup 2b, plasmid-mediated TEM-1, TEM-2, and SHV-1 were reported from the 1970s–1980s [20]. Soon after, mutated TEM and SHV ESBLs, such as TEM-3, SHV-2, and CTX-M, which efficiently degrade cefotaxime but remain sensitive to inhibition by clavulanic acid, were reported. These ESBLs belong to the Bush-Jacoby functional subgroup 2be, and since the 1990s, many variants such as BEL-1, BES-1, SFO-1, PER, and VEB were reported as members of this group [21]. TEM-30 and SHV-10, which hydrolyze penicillins and display relative resistance to clavulanic acid, sulbactam, and tazobactam, belong to Bush-Jacoby functional subgroup 2br. TEM-50 is a broad-spectrum lactamase that hydrolyzes extended-spectrum cephalosporins (oxyimino-β-lactams) and monobactams but shows resistance to clavulanic acid, sulbactam, and tazobactam. Enzymes in this group have acquired resistance to clavulanic acid, sulbactam, and tazobactam and belong to Bush-Jacoby functional subgroup 2ber [22, 23]. Class A carbenicillinases and cephalosporinases, which belong to Bush-Jacoby functional group 2, can be further classified into more minor subclasses, such as functional groups 2c, 2ce, and 2e [24]. β-lactamases, such as PSE-1 and CARB-3, belonging to Bush-Jacoby functional subgroup 2c, hydrolyze carbenicillin and are inhibited by clavulanic acid or tazobactam. A subclass of 2c, namely subclass 2ce, comprises one enzyme RTG-4 (CARB-10) that was recently identified as an extended-spectrum carbenicillinase, and hydrolyzes carbenicillin, cefepime, and cefpirome. Subclass 2ce, which includes CepA, hydrolyzes extended-spectrum cephalosporins and is inhibited by clavulanic acid or tazobactam but not aztreonam.

**Fig. 6:**
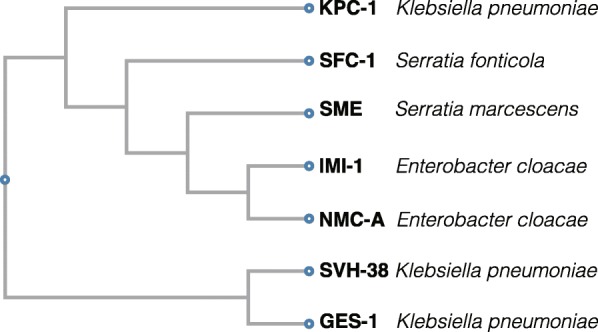
A phylogenetic tree of class A carbapenemases. A phylogenetic tree of six types of class A capbapenemases: GES (Guiana extended-spectrum β-lactamase), SME (*Serratia marcescens* enzyme), SHV-38 (sulfhydryl variable lactamase), KPC (*Klebsiella pneumoniae* carbapenemase), IMI/NMC-A (imipenemase/non-metallocarbapenemase-A), and SFC-1 (*Serratia fonticola* carbapenemase). Based on the primary sequences, the tree was generated using Clustal Omega [32–34] of GenomeNet at Kyoto University Bioinformatics Center (https://www.genome.jp/tools-bin/clustalw)

Finally, in the late 1990s, the β-lactamase classified as class A was mutated to become a carbapenemase, which degrades carbapenems [24]. As a feature of class A β-lactamases, the tetrad motif S-X-X-K, the S-D-N triad, and the K-S/T-G triad, which contain the serine of the enzyme active site in their primary sequences, are retained (Additional file 2: Figure S2) [25]. In addition, class A carbapenemases are characterized by susceptibility to inhibition by β-lactamase inhibitors such as clavulanic acid and tazobactam and are classified as Bush-Jacoby functional subgroup 2f [24]. Six types of class A carbapenemases have been reported, these include GES (Guiana extended-spectrum β-lactamase) [26], SME (*Serratia marcescens* enzyme) [27], SHV (sulfhydryl variable lactamase) [28], KPC (*Klebsiella pneumoniae* carbapenemase) [29, 30], IMI/NMC-A (imipenemase/non-metallocarbapenemase-A) [31], and SFC (*Serratia fonticola* carbapenemase) (Fig. 6) [35, 36]. The KPC-type is transmitted via a plasmid and is the most representative example of a class A carbapenemase, it also plays an important role in controlling the characteristics of carbapenem-resistant *Enterobacteriaceae* bacteria (CRE) [37]. SFC-1 and SME have been reported in *Serratia* and are closely related to KPC. SME and IMI/NMC-A are chromosomally encoded, and the GES gene *bla*GES is inserted into the class 1 integron in a plasmid of *P. aeruginosa* [38].

### Class C ESAC

The β-lactamase belonging to class C is derived from the *ampC* gene carried on the genome of many members of the *Enterobacteria* genus of the *Enterobacteriaceae* and is functionally a cephalosporinase classified into Bush-Jacoby functional group 1 [39, 40]. It is resistant to clavulanic acid, but sensitive to cephamycins, such as cefoxitin and ceftazidime. Although the expression level of AmpC is usually low, it may be induced by administration of a penicillin system or clavulanic acid and may exhibit carbapenem resistance when it is expressed in large amounts [1]. Some β-lactamases belonging to this group, including CMY, ACT, FOX, and MIR, were encoded on a plasmid [41]. The most significant concern is that variants of AmpC contribute to the reduced sensitivity to carbapenems. These β-lactamases are known as ESAC [42]. In *P. aeruginosa*, AmpC mutants have been linked to reduced sensitivity to imipenem, ceftazidime, and cefepime. Such mutants, including the plasmid-encoded CMY-10, CMY-19, and CMY-37 mutants, are classified within Bush-Jacoby functional subgroup 1e [43, 44].

The class C β-lactamases share around 40–50% identity at the primary sequence level. The amino-terminal region is the most variable, whereas the carboxyl-terminal region is relatively conserved. They share three characteristic motifs of serine-reactive β-lactamases: S-X-X-K in the enzymatic active site, Y-X-N pointing into the active site, and K-T-G forming the opposite wall of the active site (Additional file 3: Figure S3) [45].

### Class D ESBL oxacillinases (OXAs)

**Fig. 7:**
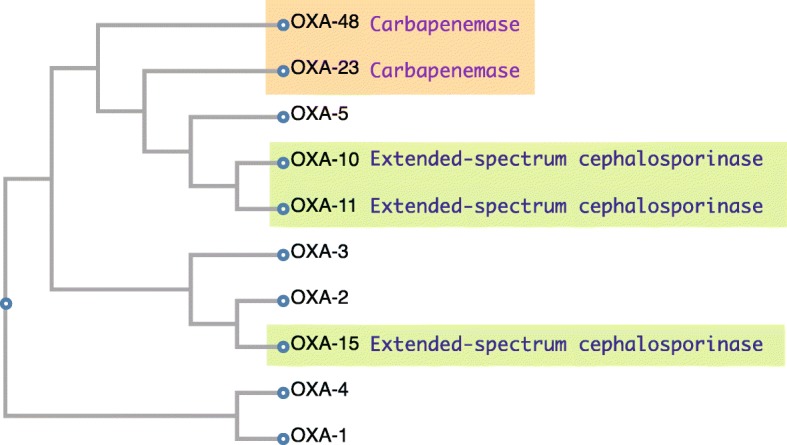
A phylogenetic tree of the class D β-lactamase OXA family (oxiacillinase). A phylogenetic tree of the OXA types of β-lactamases. OXA-10, OXA-11, and OXA-15 are recognized as extended-spectrum cephalosporinases, whereas OXA-23 and OXA-48 are recognized as carbapenem-hydrolyzing class D β-lactamases (CHDLs). Based on the primary sequences, the tree was generated using Clustal Omega [32–34] of GenomeNet at Kyoto University Bioinformatics Center (https://www.genome.jp/tools-bin/clustalw)

β-lactamases of Ambler class D, known as OXA enzymes (Fig. 7), which include OXA-1 and OXA-10, possess an active serine site similar to class A and C β-lactamases. These β-lactamases show cloxacillin- and oxacillin-hydrolyzing activity and are classified into Bush-Jacoby functional group 2d. The overall level of amino acid identity between class D and class A or class C β-lactamases is only about 16%. In β-lactamases such as OXA-11 and OXA-15, the degradation substrate is extended to extended-spectrum cephalosporins (oxyimino-β-lactams), but not carbapenems, and they are classified as Bush-Jacoby functional subgroup 2de [46, 47]. This type of β-lactamases is frequently encoded on plasmids and/or integrons allowing for their wide dissemination [48]. Carbapenem-hydrolyzing class D β-lactamases (CHDLs) are OXA enzymes that hydrolyze carbapenems and belong to Bush-Jacoby functional subgroup 2df. OXA enzymes with carbapenem-hydrolyzing activity have mainly been found on the chromosomes of *Acinetobacter baumannii* strains [49], but OXA-23 and OXA-48 have been reported on plasmids isolated from enteric bacteria [50, 51]. These OXA-type enzymes are widespread across *Acinetobacter*, *Shewanella*, *Pseudomonas*, and *Burkholderi* [48, 52]*.* At the time of writing, 790 OXA-variants could be found in public databases. Similar to class A β-lactamases, class D β-lactamases retain the tetrad motif S-X-X-K (including the serine of the enzyme active site), the Y-G-N/S triad, and the K-T-G triad (Additional file 4: Figure S4) [53].

### Class B MBL

Class B β-lactamase is a MBL that possesses the metal Zn^2+^ at the enzyme active center, compared with a serine residue in the same location for other classes of β-lactamase [54]. Class B β-lactamases hydrolyze carbapenems and belong to Bush-Jacoby functional group 3. *P. aeruginosa* carrying MBL degrades all β-lactam agents except monobactams. Since the metal is located at the enzymatic active center, its enzymatic activity is suppressed by a chelating agent, such as ethylenediaminetetraacetic acid (EDTA). The MBL gene can reside on an integron, transposon, plasmid, chromosome, or various other genetic molecules.

**Fig. 8:**
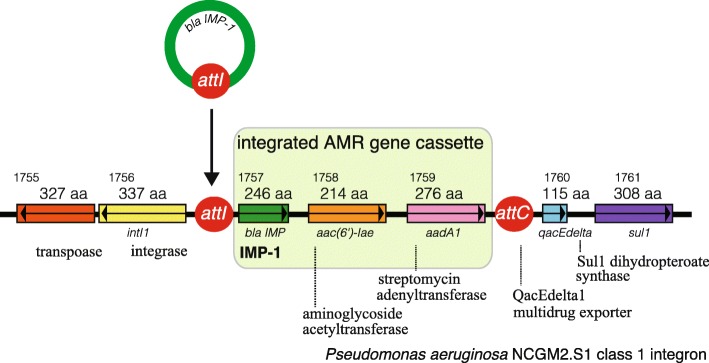
The genomic structure of the class 1 integron of *P. aeruginosa* NCGM2.S1. The *intl* gene, which encodes integrase Int1, catalyzes recombination between the *attl1* site of the integron and the *attC* site of an antimicrobial gene cassette [56]. In multidrug-resistant *P. aeruginosa* NCGM2.S1 [58], three antimicrobial gene cassettes (*bla*IMP-1, *aac(6′)-1ae*, and *aadA1*) are integrated into the region between *attI* and *attC*. The *Int*1 integrase gene, the *attI* integration site, and *qacE* partially deleted in the *sulI* gene, which encodes resistance to sulfonamide are present

The components of an integron first reported in 1989 comprised drug-resistant genes of class B MBLs and ESBLs of classes A and D [55–57]. Gene cassettes become integrated into the genome via the interaction between two recombination sites (*attI* and *attC*), and an integrase catalyzes the genetic recombination (Fig. 8). The promoter, which is located in the integrase gene upstream of the insertion site, controls the transcription of the inserted drug-resistant genes embedded in the cassette structure.

**Fig. 9:**
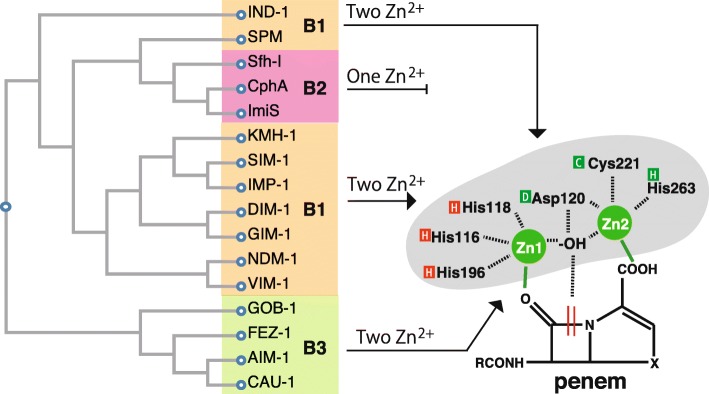
A phylogenetic tree of the class B metallo-β-lactamase family. MBLs can be classified into three subclasses (B1, B2, B3) based on their amino acid sequences. Subclasses B1 and B3 are characterized by two Zn^2+^ molecules in the enzyme active center (Zn_1_, Zn_2_), implying a broader range of substrates, whereas target substrates of subclass B2, which have a single Zn^2+^ at the active center, are narrower in range than those of classes 1 and 3 [54]. Based on the primary sequences, the tree was generated using Clustal Omega [32–34] of GenomeNet at Kyoto University Bioinformatics Center (https://www.genome.jp/tools-bin/clustalw)

To date, the carbapenem-resistant metallo-β-lactamases, imipenemase (IMP) [59], Verona integrated-encoded MBL (VIM) [60], Sao Paulo MBL (SPM) [61], Germany imipenemase (GIM) [62], New Delhi MBL (NDM) [63], and Florence imipenemase (FIM) [64] have been reported (Fig. 9, and Additional file 5: Figure S5). Among them, the IMP- and VIM-type MBLs, first discovered in the 1990s, are the major MBLs [60, 65]. New variants of IMP- and VIM-type MBLs are constantly being identified (Additional file 6: Figure S6 and Additional file 7: Figure S7). At the time of writing, 83 IMP- and 66 VIM-variants could be found in public databases. The mutations present in variants affect the spectrum of carbapenem activities, such as activity against imipenem, meropenem, and doripenem [66]. For example, IMP-6, which has only one amino acid substitution (serine to glycine at position 214) from IMP-1 (Additional file 6: Figure S6), enhanced the resistance to meropenem, and meropenem-resistant *Enterobacteriaceae*, *K. pneumoniae*, and *P. aeruginosa* carrying blaIMP-6 have been spreading in Japan since 2001 [67, 68]. Similarly, VIM-4, which has only one amino acid insertion (arginine at position 44) and one amino acid substitution (serine to arginine at position 265) from VIM-1 (Additional file 7: Figure S7), enhanced the resistance to carbapemems, and *P. aeruginosa* carrying *bla*VIM-4, which was first reported in 2002, has already been detected worldwide [69, 70]. IMP and VIM are mainly included in the integron structure and are integrated into chromosomal DNA and plasmid DNA in association with the transposon [60, 65]. NDM-1 was detected in *Klebsiella* and *E. coli* isolates from patients who returned to Sweden in 2008 following a trip to India, and the *bla*NDM-1 gene was present on the plasmid [63]. Unlike IMP and VIM, NDM was not found in the integron structure.

MBLs can be classified into three subclasses (B1, B2, B3) based on their amino acid sequence (Fig. 9) [54, 71]. The level of amino acid identity between groups was as low as 20% or less. Subclass B1 includes IMP, VIM, NDM, and SPM, and subclass B3 includes CAU-1, GOB-1, and FEZ-1, both of which are characterized by the presence of two Zn^2+^ molecules in the enzyme active center (Zn_1_, Zn_2_), degradation of a broader range of substrates, and classification into Bush-Jacoby functional subgroup 3a [54]. The binding site for Zn_1_ of the B1 enzyme involves three histidines (His116, His118, and His196) [72]. The binding site for Zn_2_ of the B1 enzyme consists of aspartate, cystine, and histidine (DCH site, Asp-120, Cys-221, His-263) (Additional file 5: Figure S5) [72], whereas the target degradation substrates for CphA, Sfh-I, and ImiS, which are MBLs of subclass B2 having one Zn^2+^ at the active center, are narrow and these MBLs are classified into Bush-Jacoby functional subgroup 3b [73, 74].

## Summary

**Fig. 10:**
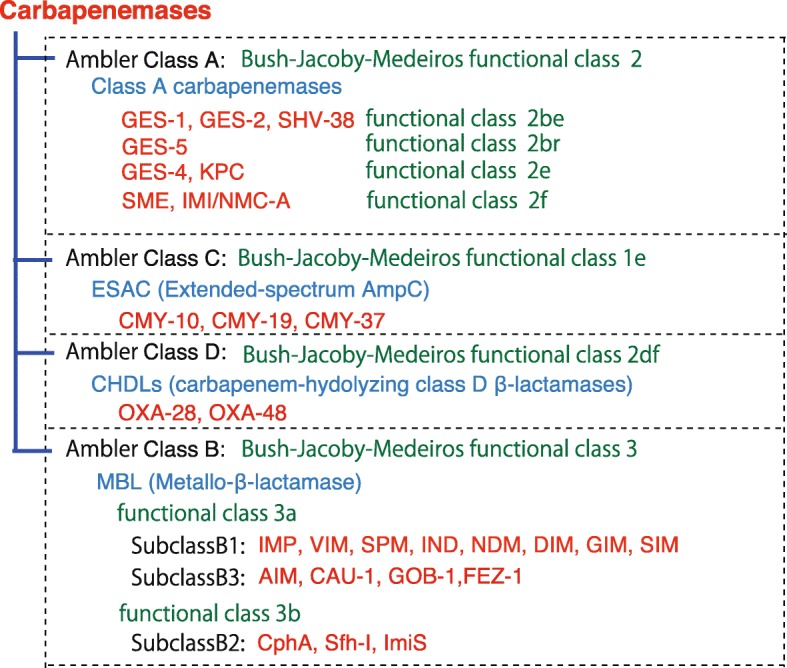
The classification of carbapenemases. Carbapenemases is represented in all classes, A to D, of the Ambler classification system [16]. Functional classification, using the Bush-Jacobi-Medeiros method [17, 18], indicated that the class A carbapenemases were represented by GES and KPC. The β-lactamases belonging to class C, which function as cephalosporinases, are encoded by the *AmpC* gene carried on the chromosome of many Enterobacteriaceae. ESAC enzymes are known as ESACs. In class D, OXA enzymes, which were originally oxacillinases, have mutated to become CHDLs. Class B β-lactamases are characterized by possessing a metal Zn^2+^ as the enzyme activity center. IMP- and VIM-type β-lactamases are the main MBLs that fit into the integron structure. MBLs can be classified into three subclasses (B1, B2, B3) based on their amino acid sequence. Subclasses B1 and B3 are characterized by two zinc molecules (Zn_1_, Zn_2_) in the enzyme active center, demonstrating more extensive substrate degradation, while subclass B2 has a single Zn^2+^ at the active center and displays a narrower spectrum

ESBLs and carbapenemases are represented in all classes, A to D, in the Ambler classification system. Class A carbapenemases, represented by GES and KPC, are encoded on plasmids and are most frequently detected in *P. aeruginosa* and *Klebsiella*. As a characteristic feature, they exert an inhibitory effect by β-lactamase inhibitors such as clavulanic acid and tazobactam. The β-lactamases belonging to class C, including ESACs, are encoded by the *ampC* gene carried on the chromosome of many *Enterobacteriaceae*, and function as cephalosporinases. In class D, the OXA enzymes, which were originally oxacillinases, have mutated into CHDLs. These enzymes have also been found to be encoded on the chromosome of carbapenem-resistant *A. baumannii* and on the plasmids of intestinal bacteria such as OXA-23 and OXA-48. Class B β-lactamases are characterized as having a metal Zn^2+^ in their enzyme activity center. *P. aeruginosa* carrying an MBL degrades all β-lactam agents, except monobactams. The MBL gene is encoded on an integron, transposon, plasmid, chromosome, or various other genetic molecules. Among them, the IMP- and VIM-type enzymes, first discovered in the 1990s, are the main MBLs that fit into the integron structure. MBLs can be classified into three subclasses (B1, B2, B3) based on their amino acid sequence. Subclass B1 includes IMP, VIM, and NDM. Subclass B3 is characterized by the presence of two zinc molecules (Zn_1_, Zn_2_) in the enzyme active center, demonstrating more extensive substrate degradation, whereas subclass B2 has a single Zn^2+^ in the active center and shows a narrow spectrum of substrates. Figure 10 summarizes carbapenemases of the Ambler classification system [16], including the functional information based on Bush-Jacobi-Medeiros method [17, 18]. Table 1 summarizes the functional classification scheme based on the 1995 proposal by Bush-Jacobi-Medeiros [18] that was updated by Bush-Jacobi [17] in 2010. Together with the Ambler molecular structure classification, antibiotic substrates hydrolyzed by classified β-lactamases and the profiles to β-lactamase inhibitors (clavulanic acid, sulbactam, and tazobactam) are also listed in this table.

**Table 1:** Classification schemes for bacterial β-lactamases by Bush-Jacoby [17, 18]

Bush-Jacoby*		Ambler	Enzymatic center	Enzymes	Distinctive substrate	Characteristic(s)
1	1	C	Ser	AmpC, CMY-1, ACT-1, FOX-1, MIR-1	Cephalosporins	Resistance to CA and TZB
	1e			GC1, CMY-10, CMY-19, CMY-37	Ceftazidime, oxyimino-β-lactams ESAC	Resistance to CA and TZB reduced sensitivity to carbapenems
2	2a	A		PC1	Penicillins	Inhibited by CA, TZB
	2b			TEM-1, TEM-2, SHV-1	Penicillins, early cephalosporins	Inhibited by CA, TZB
	2be			TEM-3, SHV-2, CTX-M-15, PER-1, VEB-1	ESCP, monobactams	Inhibited by CA, TZB
	2br			TEM-30, SHV-10	Penicillins	Resistance to CA, SB and TZB
	2ber			TEM-50	ESCP, monobactams	Resistance to CA, SB and TZB
	2c			PSE-1, CARB-3	Carbenicillin	Inhibited by CA, TZB
	2ce			RTG-4	Carbenicillin, cefepime	Inhibited by CA, TZB
	2d	D		OXA-1, OXA-10	Cloxacillin	Variable to CA, TZB
	2de			OXA-11, OXA-15	ESCP, oxyimino-β-lactams	
	2df			OXA-23, OXA-48	Cloxacillin, oxacillin, carbapenems	
	2e	A		CepA	ESCP	Inhibited by CA but not aztreonam
	2f			KPC-2, IMI-1, SME-1	Carbapenems, oxyimino-β-lactams, cephamycins	Variable to CA, TZB
3	3a	B1	Zn^2+^	IMP-1, VIM-1,CcrA, IND-1	Carbapenems	Resistance to CA, SB and TZB Inhibited by monobactams
	3b	B3		L1, CAU-1, GOB-1, FEZ-1		
	3c	B2		CphA, Sfh-1		Resistance to CA, SB and TZB

*CA* clavulanic acid, *SB* sulbactam, *TZB* tazobactam, *ESCP* Extended-spectrum cephalosporins

*Updated in 2010 [17]

The following is a list of important issues that clinicians should keep in mind during the daily management of infections:

1. Basic science: PBPs (Fig. 2), which act as adenyl-alanine endopeptidases, enzymatically target an alanyl-alanine of the peptidoglycan pillar structure and catabolize the cross-links between the peptidoglycan layers (Fig. 1). β-lactams (Fig. 4) mimic the alanyl-alanine structure (Fig. 3) and inhibit the enzymatic action of PBPs. β-lactamases share the enzyme active site motifs with the PBP family, indicating that β-lactamases and PBPs have co-evolved (Additional file 1: Figure S1).
2. Classification: β-lactamases have been classified in two ways: one method is based on the molecular structure classification of Ambler, and the other method is based on the functional classification of Bush-Jacobi-Medeiros (Fig. 5). Understanding both structural and functional classifications is important for clinicians in choosing the most appropriate antimicrobial agent (Table 1).
3. ESBLs and carbapenemases: ESBLs and carbapenemases are represented in all classes, A to D, in the Ambler classification system (Figs. 5 and 10). Carbapenemases in all classes have been found on both plasmids and chromosomal genomes. The *bla*GES gene of class A (Fig. 6 and Additional file 2: Figure S2), *bla*OXA of class D (Fig. 7 and Additional file 4: Figure S4), and the MBL genes of class B (Fig. 9 and Additional file 5: Figure S5) are frequently encoded in an integron (Fig. 8). The extended-spectrum β-lactamases belonging to class C are encoded by the *ampC* gene usually carried on the chromosome (Additional file 3: Figure S3).
4. Diversity: The accumulation of small mutations in the primary amino acid sequences of β-lactamases ultimately extends the substrate spectrum. Clinicians can gain insight into how the use of antimicrobial agents may affect the molecular mutation of lactamases by analyzing the alignment of amino acid sequences of specific MBLs, such as IMP (Additional file 6: Figure S6) and VIM (Additional file 7: Figure S7).
5. Diagnostic: Finally, the characterization of antimicrobial resistance is critical for the classification of β-lactam resistance. For more information, please refer to references on the detection of β-lactamase-mediated resistance [75–78], because this is beyond the scope of this review. In brief, microbiological tests such as the Etest [76], the Clinical and Laboratory Standards Institute (CLSI) method [77] and the Double Disc Synergy Test (DDST) [78] can identify the phenotypes of ESBL, MBL, and ESAC. Novel multiplex polymerase chain reaction (PCR)-based ORF typing (POT) methods that are routinely used in clinical laboratories can identify the prevalent ESBL and carbapenemase genes, such as SHV, GES, TEM, CTX-M, and KPC of class A, NDM, IMP, and VIM of class B, and OXA-48 of class D in *A. baumannii* [79], *P. aeruginosa* clones [80], and other pathogenic bacteria. Antimicrobial gene cassettes, which are sometimes integrated into the integron structure via the interaction between two recombination sites (*attI* and *attC*), can be identified by PCR amplification with a primer set specific for the consensus sequence (CS) regions (5′-CS and 3′-CS) located upstream and downstream of the insertional site and subsequent DNA sequence analysis [81–84].

## Conclusion

Various ESBLs and carbapenemases belonging to one of four molecular classes have propagated and are being detected among bacteria worldwide, suggesting that careful detection and monitoring are vital when critical care clinicians are treating infections caused by ESBL- and carbapenem-resistant bacteria. For the classification of β-lactamases, the Ambler method of molecular structure classification [16] is simple and effective at organizing the various ESBLs and carbapenemases, but functional classification using the Bush-Jacobi-Medeiros method [17, 18] is also important for clinicians faced with treating patients in a critical condition due to ESBL- and carbapenem-resistant bacterial infections.

## Supplementary information

**Additional file 1: Figure S1.** The similarities in the structures of class A β-lactamases and penicillin-binding protein (PBP).

**Additional file 2: Figure S2.** The alignment of class A carbapenemases based on their primary sequences.

**Additional file 3: Figure S3.** The alignment of class C β-lactamases.

**Additional file 4: Figure S4.** Alignment of the class D β-lactamase OXA family (oxacillinase).

**Additional file 5: Figure S5**. Alignment of the class B metallo-β-lactamase family and their zinc enzymatic active center.

**Additional file 6: Figure S6.** The alignment of primary sequences of the class B metallo-β-lactamase IMP family.

**Additional file 7: Figure S7.** The alignment of primary sequences of the class B metallo-β-lactamase VIM family.

## Data Availability

All data generated or analyzed during this study are included in this published article and its supplementary information files.

## Abbreviations

CHDL: Carbapenem-hydrolyzing class D β-lactamases
CLSI: Clinical and Laboratory Standards Institute
CPE: Carbapenemase-producing *Enterobacteriaceae*
CS: Consensus sequence
DDST: Double disc synergy test
EDTA: Ethylenediaminetetraacetic acid
ESAC: Extended-spectrum AmpC
ESBL: Extended-spectrum β-lactamase
FIM: Florence imipenemase
GES: Guiana extended-spectrum β-lactamase
GIM: Germany imipenemase
HMW-PBP: High molecular weight penicillin-binding protein
IMI/NMC-A: Imipenemase/non-metallocarbapenemase-A
IMP: Imipenemase
KPC: *Klebsiella pneumoniae* carbapenemase
LMW-PBP: High molecular weight penicillin-binding protein
MBL: Metallo-β-lactamase
MDRP: Multidrug-resistant *Pseudomonas aeruginosa*
MRSA: Methicillin-resistant *Staphylococcus aureus*
NAG: *N*-acetylglucosamine
NAM: *N*-acetylmuramic acid
NDM: New Delhi metallo-β-lactamase
PBP: Penicillin-binding protein
PCR: Polymerase chain reaction
PDRP: Pandrug-resistant *Pseudomonas aeruginosa*
POT: PCR-based ORF typing
SFC: *Serratia fonticola* carbapenemase
SHV: Sulfhydryl variable lactamase
SME: *Serratia marcescens* enzyme
SPM: Sao Paulo metallo-β-lactamase
VIM: Verona integrated-encoded MBL
XDRP: Extensively drug-resistant *Pseudomonas aeruginosa*
