# Supplementary material for: Molecular diversity of extended-spectrum β-lactamases and carbapenemases, and antimicrobial resistance
Source: J Intensive Care. 2020 Jan 28;8:13. doi: 10.1186/s40560-020-0429-6 (PMC6988205; doi:10.1186/s40560-020-0429-6)
Supplement: Supplementary file 6 — Additional file 6: Figure S6. The alignment of primary sequences of the class B metallo-β-lactamase IMP family. [file 40560_2020_429_MOESM6_ESM.pdf]

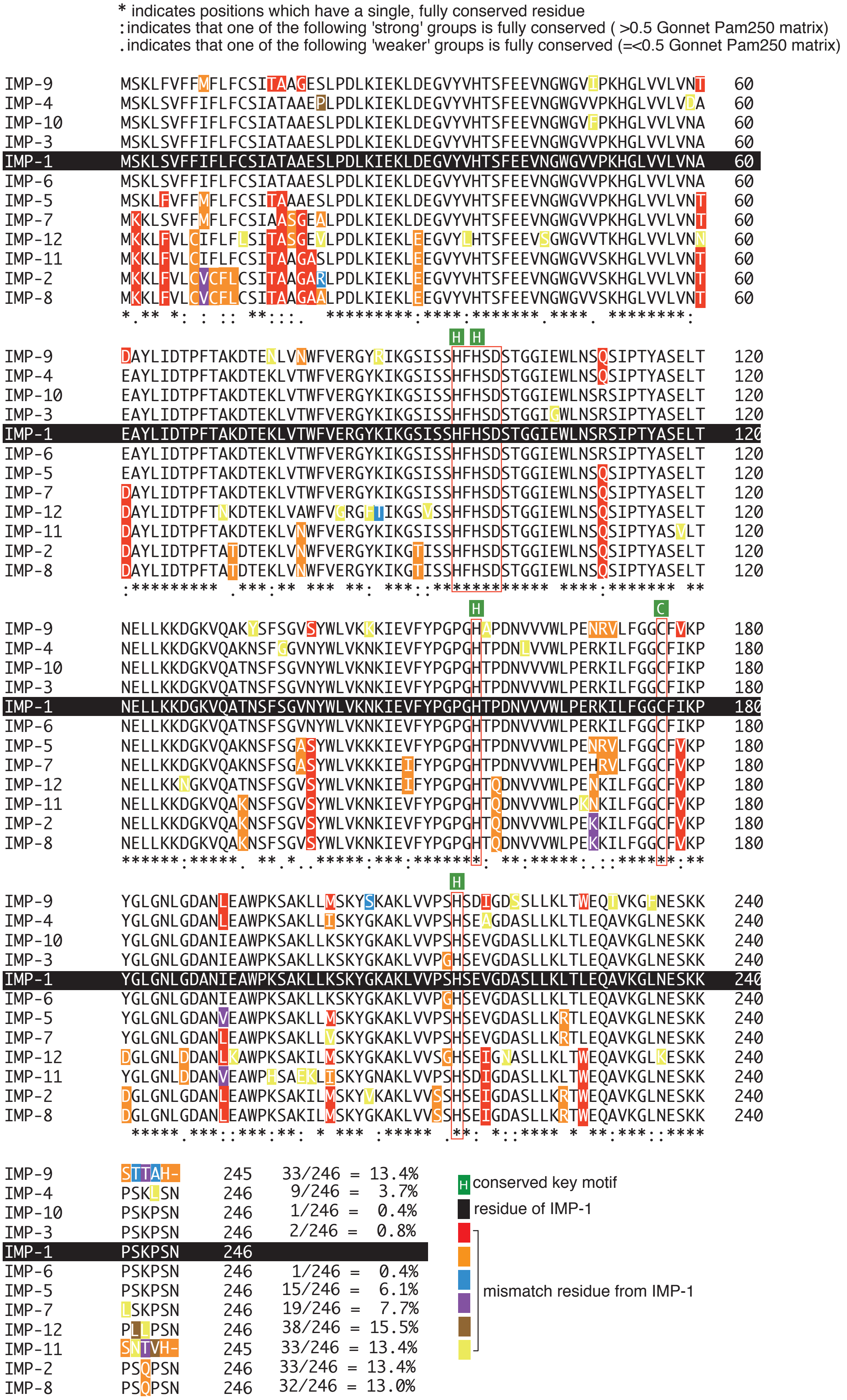

Supplemental Fig. 6. The alignment of primary sequences of the class B metallo-β-lactamase IMP family

Primary sequence variations as evidenced by the alignment of IMP-type MBLs. The binding site for Zn^{2+} at the enzymatic active site involves three histidines at the amino-terminal side. Based on the primary sequences, the alignment was generated using Clustal Omega [32, 33, 34] of GenomeNet at Kyoto University Bioinformatics Center (https://www.genome.jp/tools-bin/clustalw).
