# Supplementary material for: Molecular diversity of extended-spectrum β-lactamases and carbapenemases, and antimicrobial resistance
Source: J Intensive Care. 2020 Jan 28;8:13. doi: 10.1186/s40560-020-0429-6 (PMC6988205; doi:10.1186/s40560-020-0429-6)
Supplement: Supplementary file 7 — Additional file 7: Figure S7. The alignment of primary sequences of the class B metallo-β-lactamase VIM family. [file 40560_2020_429_MOESM7_ESM.pdf]

* indicates positions which have a single, fully conserved residue
: indicates that one of the following 'strong' groups is fully conserved (>0.5 Gonnet Pam250 matrix)
. indicates that one of the following 'weaker' groups is fully conserved (=<0.5 Gonnet Pam250 matrix)

```
VIM-7     MF-QIRSFLVGISAFVMAVLGSAAYSAQPGGEYPTVDDIPVGEVRLYKIGDGVWSHIATQ   59
VIM-3     MFKLLSKLLVYLTASIMAIASPLAFSVDSSGEYPTVSEIPVGEVRLYQIADGVWSHIATK   60
VIM-6     MFKLLSKLLVYLTASIMAIASPLAFSVDSSGEYPTVSEIPVGEVRLYQIADGVWSHIATR   60
VIM-11    MFKLLSKLLVYLTASIMAIASPLAFSVDSSGEYPTVSEIPVGEVRLYQIADGVWSHIATQ   60
VIM-10    MFKLLSKLLVYLTASIMAIASPLAFSVDSSGEYPTVSEIPVGEVRLYQIADGVWSHIATQ   60
VIM-2     MFKLLSKLLVYLTASIMAIASPLAFSVDSSGEYPTVSEIPVGEVRLYQIADGVWSHIATQ   60
VIM-8     MFKLLSKLLVYLTASIMAIASPLAFSVDSSGEYPTVSEIPVGEVRLYQIADGVWSHIATQ   60
VIM-9     MFKLLSKLLVYLTASIMAIASPLAFSVDSSGEYPTVSEIPVGEVRLYQIADGVWSHIATQ   60
VIM-12    MLKVISSLLVYMTASVMAVASPLAHSGEPSGEYPTVNEIPVGEVRLYQIADGVWSHIATQ   60
VIM-1     MLKVISSLLVYMTASVMAVASPLAHSGEPSGEYPTVNEIPVGEV-LYQIADGVWSHIATQ   59
VIM-4     MLKVISSLLVYMTASVMAVASPLAHSGEPSGEYPTVNEIPVGEVRLYQIADGVWSHIATQ   60
VIM-5     MLKVISSLLVYMTASVMAVASPLAHSGEPSGEYPTVNEIPVGEVRLYQIADGVWSHIATQ   60
          *:  : .:** ::* :**: .  *.* : .******.:****** **:*.*********:

                                                            H H
VIM-7     KLGDTVYSSNGLIVRDADELLLIDTAWGAKNTVALLAEIEKQIGLPVTRSISTHFHDDRV   119
VIM-3     SFDGAVYPSNGLIVRDGDELLLIDTAWGAKNTAALLAEIEKQIGLPVTRAVSTHFHDDRV   120
VIM-6     SFDGAVYPSNGLIVRDGDELLLIDTAWGAKNTAALLAEIEKQIGLPVTRAVSTHFHDDRV   120
VIM-11    SFDGAVYPSNGLIVRDGDELLLIDTAWGAKNTAALLAEIEKQIGLPVTRAVSTHFHDDRV   120
VIM-10    SFDGAVYPSNGLIVRDGDELLLIDTAWGAKNTAALLAEIEKQIGLPVTRAVSTHFHDDRV   120
VIM-2     SFDGAVYPSNGLIVRDGDELLLIDTAWGAKNTAALLAEIEKQIGLPVTRAVSTHFHDDRV   120
VIM-8     SFDGAVYPSNGLIVRDGDELLLIDTAWGAKNTAALLAEIEKQIGLPVTRAVSTHFHDDRV   120
VIM-9     SFDGAVYPSNGLIVRDGDELLLIDTAWGAKNTAALLAEIEKQIGLPVTRAVSTHFHDDRV   120
VIM-12    SFDGAVYPSNGLIVRDGDELLLIDTAWGAKNTAALLAEIEKQIGLPVTRAVSTHFHDDRV   120
VIM-1     SFDGAVYPSNGLIVRDGDELLLIDTAWGAKNTAALLAEIEKQIGLPVTRAVSTHFHDDRV   119
VIM-4     SFDGAVYPSNGLIVRDGDELLLIDTAWGAKNTAALLAEIEKQIGLPVTRAVSTHFHDDRV   120
VIM-5     SFDGAVYPSNGLIVRDGDELLLIDTAWGAKNTAALLAEIEKQIGLPVTRAVSTHFHDDRV   120
          .:..:** ********.***************.****************::*********

                                                                H
VIM-7     GGVDVLRAAGVATYTSPLTRQLAEAAGNEVPAHSLKALSSSGDVVRFGPVEVFYPGAAHS   179
VIM-3     GGVDVLRAAGVATYASPSTRRLAEVEGSEIPTHSLEGLSSSGDAVRFGPVELFYPGAAHS   180
VIM-6     GGVDVLRAAGVATYASPSTRRLAEVEGSEIPTHSLEGLSSSGDAVRFGPVELFYPGAAHS   180
VIM-11    GGVDVLRAAGVATYASPSTRRLAEVEGSEIPTHSLEGLSSSGDAVRFGPVELFYPGAAHS   180
VIM-10    GGVDVLRAAGVATYASPSTRRLAEVEGNEIPTHSLEGLSSSGDAVRFGPVELFYPGAAHS   180
VIM-2     GGVDVLRAAGVATYASPSTRRLAEVEGNEIPTHSLEGLSSSGDAVRFGPVELFYPGAAHS   180
VIM-8     GGVDVLRAAGVATYASPSARRLAEVEGNEIPTHSLEGLSSSGDAVRFGPVELFYPGAAHS   180
VIM-9     GGVDVLRAAGVATYASPSIRRLAEVEGNEIPTHSLEGLSSSGDAVRFGPVELFYPGAAHS   180
VIM-12    GGVDVLRAAGVATYASPSTRRLAEAEGNEIPTHSLEGLSSSGDAVRFGPVELFYPGAAHS   180
VIM-1     GGVDVLRAAGVATYASPSTRRLAEAEGNEIPTHSLEGLSSSGDAVRFGPVELFYPGAAHS   179
VIM-4     GGVDVLRAAGVATYASPSTRRLAEAEGNEIPTHSLEGLSSSGDAVRFGPVELFYPGAAHS   180
VIM-5     GGVDVLRKAGVATYASPSTRRLAEAEGNEIPTHSLEGLSSSGDAVRFGPVELFYPGAAHS   180
          ******* ******:**  *:***. *.*:*:***:.******.*******:********

                           H                                         H
VIM-7     GDNLVVYVPAVRVLFGGCAVHEASRESAGNVADANLAEWPATIKRIQQRYPEAEVVIPGH   239
VIM-3     TDNLVVYVPSASVLYGGCAIYELSRTSAGNVADADLAEWPTSIERIQQHYPEAQFVIPGH   240
VIM-6     TDNLVVYVPSASVLYGGCAIYELSRTSAGNVADADLAEWPTSIERIQQHYPEAQFVIPGH   240
VIM-11    TDNLVVYVPSASVLYGGCAIYELSRTSAGNVADADLAEWPTSIERIQQHYPEAQFVIPGH   240
VIM-10    TDNLVVYVPSASVLYGGCAIYELSRTSAGNVADADLAEWPTSIERIQQHYPEAQYVIPGH   240
VIM-2     TDNLVVYVPSASVLYGGCAIYELSRTSAGNVADADLAEWPTSIERIQQHYPEAQFVIPGH   240
VIM-8     TDNLVVYVPSASVLYGGCAIYELSRTSAGNVADADLAEWPTSIERIQQHYPEAQFVIPGH   240
VIM-9     TDNLVVYVPSASVLYGGCAIYELSRTSAGNVADADLAEWPTSIERIQQHYPEAQFVIPGH   240
VIM-12    TDNLVVYVPSANVLYGGCAVHELSSTSAGNVADADLAEWPTSIERIQQHYPEAQFVIPGH   240
VIM-1     TDNLVVYVPSANVLYGGCAVHELSSTSAGNVADADLAEWPTSVERIQKHYPEAEVVIPGH   239
VIM-4     TDNLVVYVPSANVLYGGCAVHELSRTSAGNVADADLAEWPTSVERIQKHYPEAEVVIPGH   240
VIM-5     TDNLVVYVPSANVLYGGCAVLALSRTSAGNVADADLAEWPTSVERIQKHYPEAEVVIPGH   240
           ********:. **:** *:   *  ********:*****::::***::****: *****

VIM-7     GLPGGLELLQHTTNVVKTHKVRPVAE                265     62/266 = 23.3%
VIM-3     GLPGGLDLLKHTTNVVKAHTNRSVVE                266     27/266 = 10.2%
VIM-6     GLPGGLDLLKHTTNVVKAHTNRSVVE                266     27/266 = 10.2%
VIM-11    GLPGGLDLLKHTTNVVKAHTNRSVVE                266     26/266 =  9.8%
VIM-10    GLPGGLDLLKHTTNVVKAHTNRSVVE                266     25/266 =  9.4%
VIM-2     GLPGGLDLLKHTTNVVKAHTNRSVVE                266     25/266 =  9.4%
VIM-8     GLPGGLDLLKHTTNVVKAHTNRSVVE                266     26/266 =  9.8%
VIM-9     GLPGGLDLLKHTTNVVKAHTNRSVVE                266     26/266 =  9.8%
VIM-12    GLPGGLDLLKHTTNVVKAHTNRSVVE                266      8/266 =  3.0%
VIM-1     GLPGGLDLLQHTANVVKAHKNRSVAE                265
VIM-4     GLPGGLDLLQHTANVVKAHKNRSVAE                266      2/266 =  0.8%
VIM-5     GLPGGLDLLQHTANVVTAHKNRSVAE                266      5/266 =  1.9%
          ******:**:**:***.:*. * *.*
```

H conserved key motif
residue of IMP-1
mismatch residue from VIM-1

Supplemental Fig. 7. The alignment of primary sequences of the class B metallo-β-lactamase VIM family

Primary sequence variations as evidenced by the alignment of VIM-type MBLs. The binding site for Zn^{2+} at the enzymatic active site involves three histidines at the amino-terminal side. Based on the primary sequences, the alignment was generated using Clustal Omega [32, 33, 34] of GenomeNet at Kyoto University Bioinformatics Center (https://www.genome.jp/tools-bin/clustalw).
