# Supplementary material for: Molecular diversity of extended-spectrum β-lactamases and carbapenemases, and antimicrobial resistance
Source: J Intensive Care. 2020 Jan 28;8:13. doi: 10.1186/s40560-020-0429-6 (PMC6988205; doi:10.1186/s40560-020-0429-6)
Supplement: Supplementary file 3 — Additional file 3: Figure S3. The alignment of class C β-lactamases. [file 40560_2020_429_MOESM3_ESM.pdf]

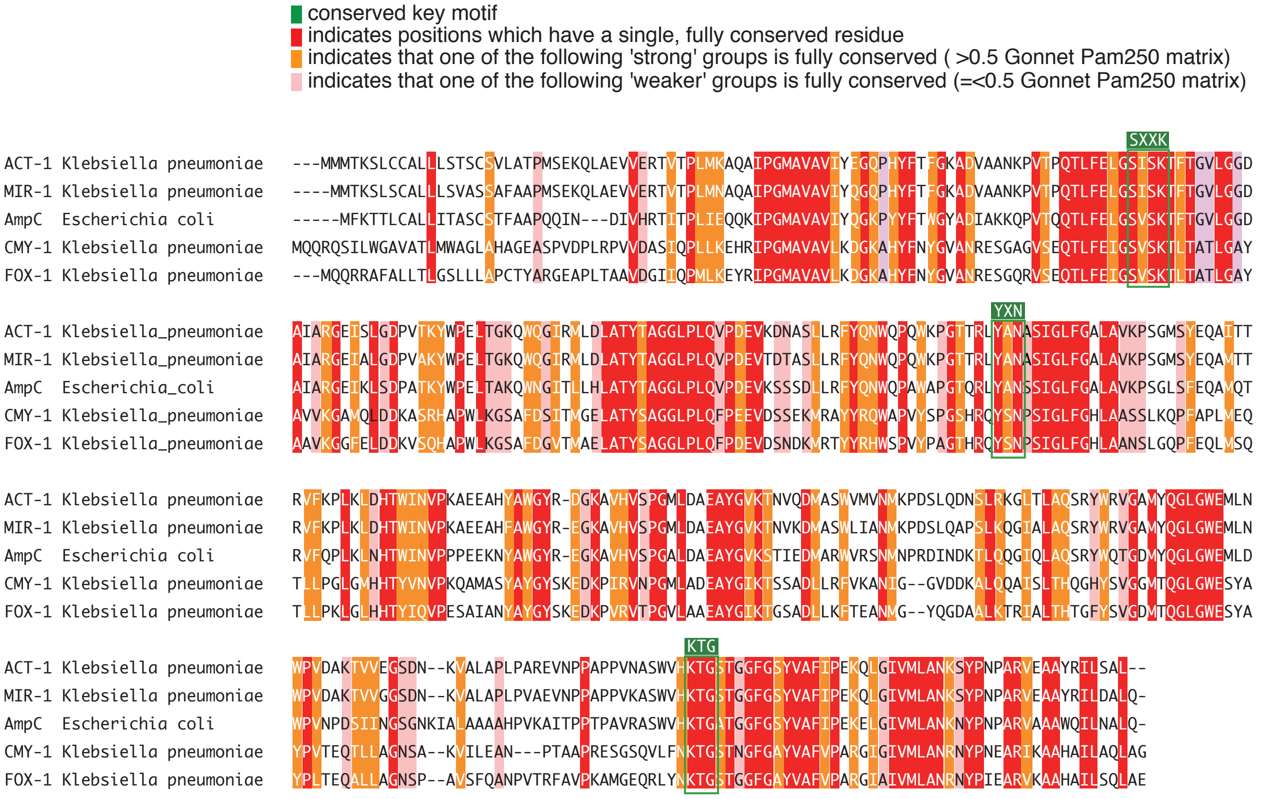

Supplemental Fig. 3. The alignment of class C β-lactamases

Class C β-lactamases share the three characteristic motifs of serine-reactive β-lactamases: S-X-X-K in the enzymatic active site, Y-X-N pointing into the active site, and K-T-G forming the opposite wall of the active site. Based on the primary sequences, the alignment was generated using Clustal Omega [32, 33, 34] of GenomeNet at Kyoto University Bioinformatics Center (https://www.genome.jp/tools-bin/clustalw).
