# Supplementary material for: Molecular diversity of extended-spectrum β-lactamases and carbapenemases, and antimicrobial resistance
Source: J Intensive Care. 2020 Jan 28;8:13. doi: 10.1186/s40560-020-0429-6 (PMC6988205; doi:10.1186/s40560-020-0429-6)
Supplement: Supplementary file 4 — Additional file 4: Figure S4. Alignment of the class D β-lactamase OXA family (oxacillinase). [file 40560_2020_429_MOESM4_ESM.pdf]

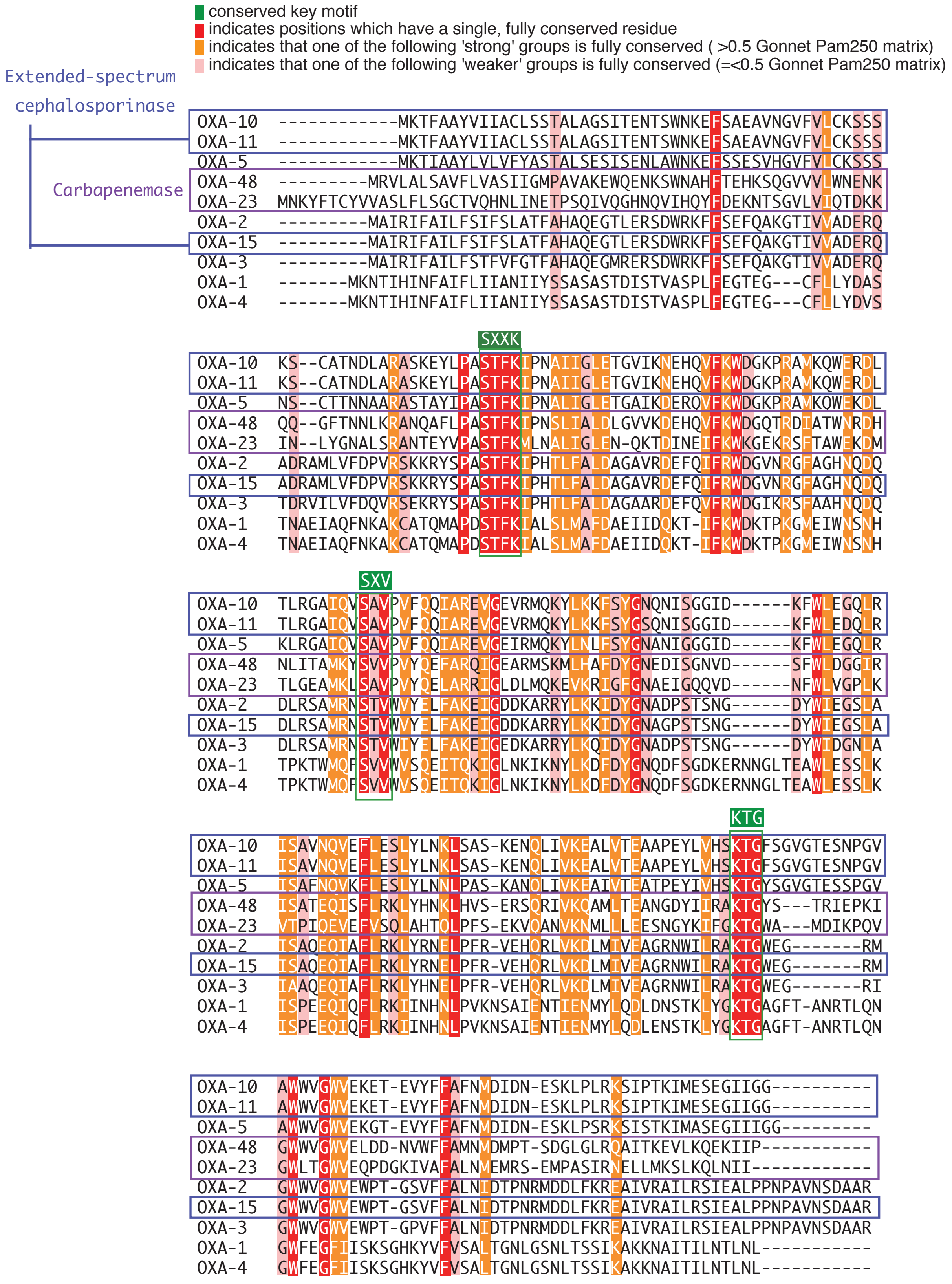

Supplemental Fig. 4. Alignment of the class D β-lactamase OXA family (oxacillinase)
OXA-10, OXA-11, and OXA-15 are recognized as extended-spectrum cephalosporinases, whereas OXA-23 and OXA 48 are recognized as CHDLs. Class D β-lactamases retain the tetrad motif S-X-X-K (including the serine of the enzyme active site), the Y-G-N/S triad, and the K-S/T-G triad. Based on the primary sequences, the alignment was generated using Clustal Omega [32, 33, 34] of GenomeNet at Kyoto University Bioinformatics Center (https://www.genome.jp/tools-bin/clustalw).
