# Supplementary material for: Molecular diversity of extended-spectrum β-lactamases and carbapenemases, and antimicrobial resistance
Source: J Intensive Care. 2020 Jan 28;8:13. doi: 10.1186/s40560-020-0429-6 (PMC6988205; doi:10.1186/s40560-020-0429-6)
Supplement: Supplementary file 5 — Additional file 5: Figure S5. Alignment of the class B metallo-β-lactamase family and their zinc enzymatic active center. [file 40560_2020_429_MOESM5_ESM.pdf]

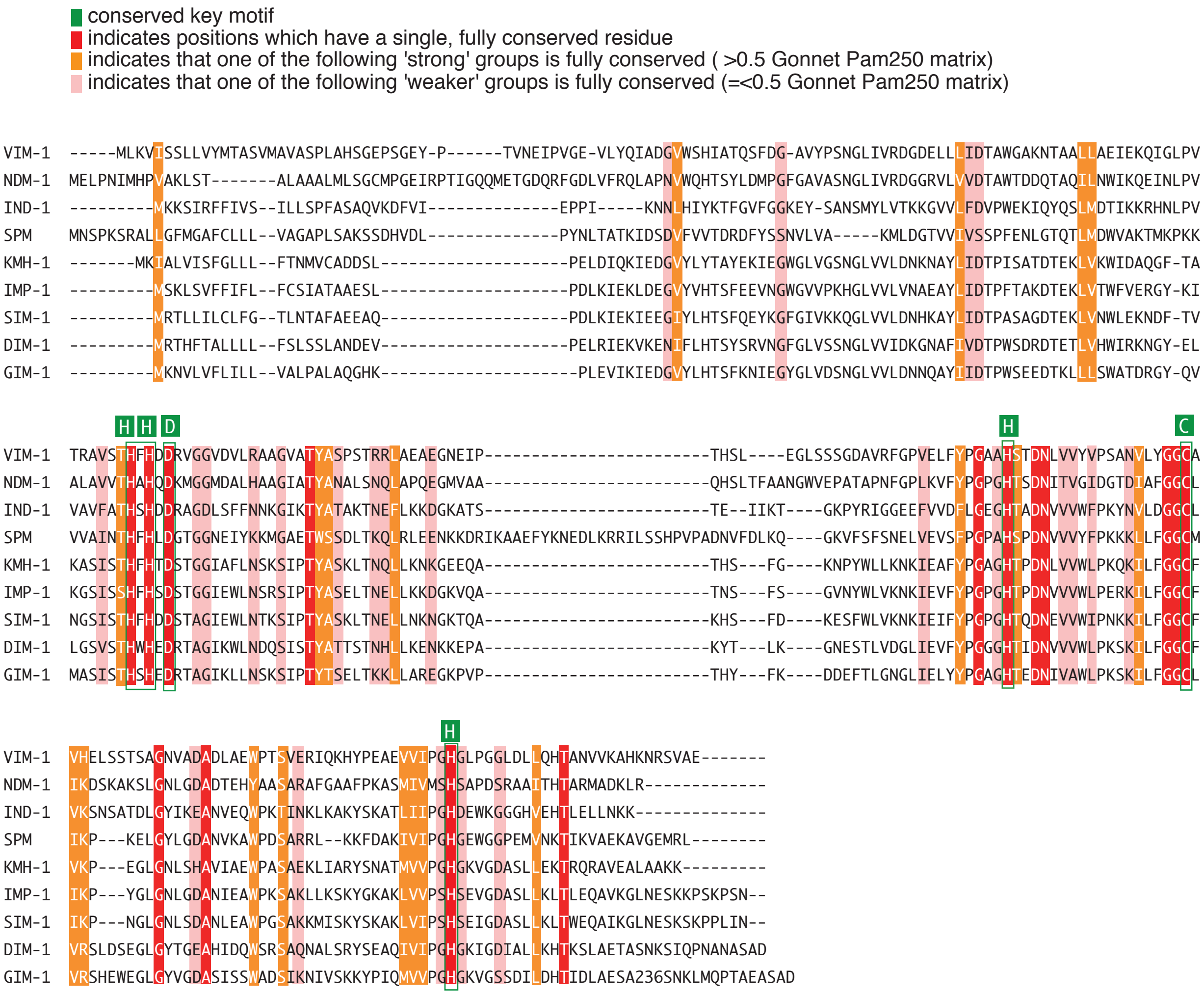

Supplemental Fig. 5. Alignment of the class B metallo-β-lactamase family and their zinc enzymatic active center

The level of amino acid identity between groups was as low as 20% or less. As a feature of class B β-lactamases, the binding site for Zn2+ at the enzymatic active site involves three histidines (His116, His118, and His196). Based on the primary sequences, the alignment was generated using Clustal Omega [32, 33, 34] of GenomeNet at Kyoto University Bioinformatics Center (https://www.genome.jp/tools-bin/clustalw).
