# Supplementary material for: Molecular diversity of extended-spectrum β-lactamases and carbapenemases, and antimicrobial resistance
Source: J Intensive Care. 2020 Jan 28;8:13. doi: 10.1186/s40560-020-0429-6 (PMC6988205; doi:10.1186/s40560-020-0429-6)
Supplement: Supplementary file 1 — Additional file 1: Figure S1. The similarities in the structures of class A β-lactamases and penicillin-binding protein (PBP). [file 40560_2020_429_MOESM1_ESM.pdf]

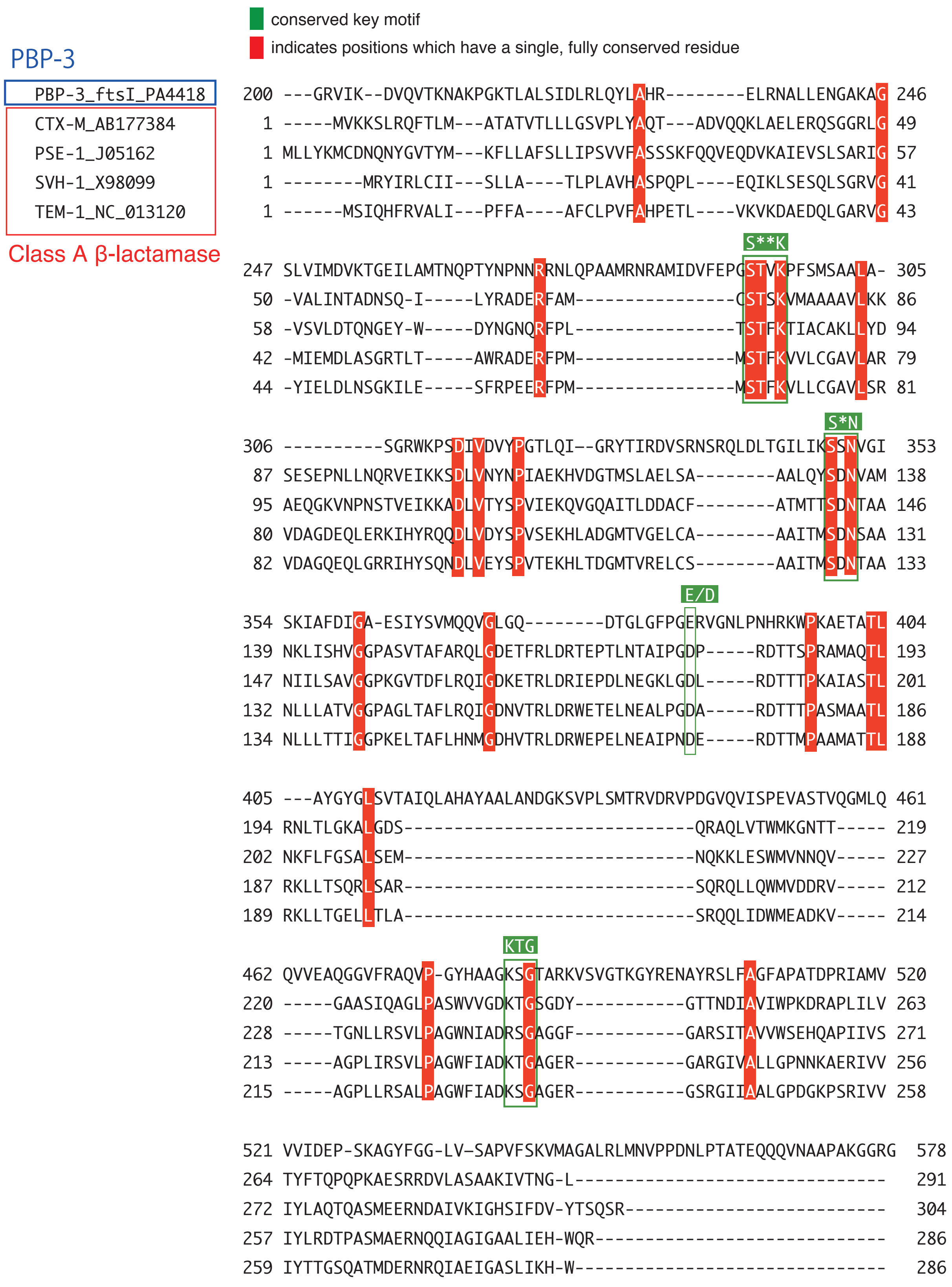

Supplemental Fig. 1. The similarities in the structures of class A β-lactamases and penicillin-binding protein (PBP)

The enzyme active site motifs S-X-X-K, S-X-N, and K-S/T-G in the primary sequence of class A β-lactamases also exist in PBP3. Based on the primary sequences, the alignment was generated using Clustal Omega [32, 33, 34] of GenomeNet at Kyoto University Bioinformatics Center (https://www.genome.jp/tools-bin/clustalw).
