# Supplementary material for: Molecular diversity of extended-spectrum β-lactamases and carbapenemases, and antimicrobial resistance
Source: J Intensive Care. 2020 Jan 28;8:13. doi: 10.1186/s40560-020-0429-6 (PMC6988205; doi:10.1186/s40560-020-0429-6)
Supplement: Supplementary file 2 — Additional file 2: Figure S2. The alignment of class A carbapenemases based on their primary sequences. [file 40560_2020_429_MOESM2_ESM.pdf]

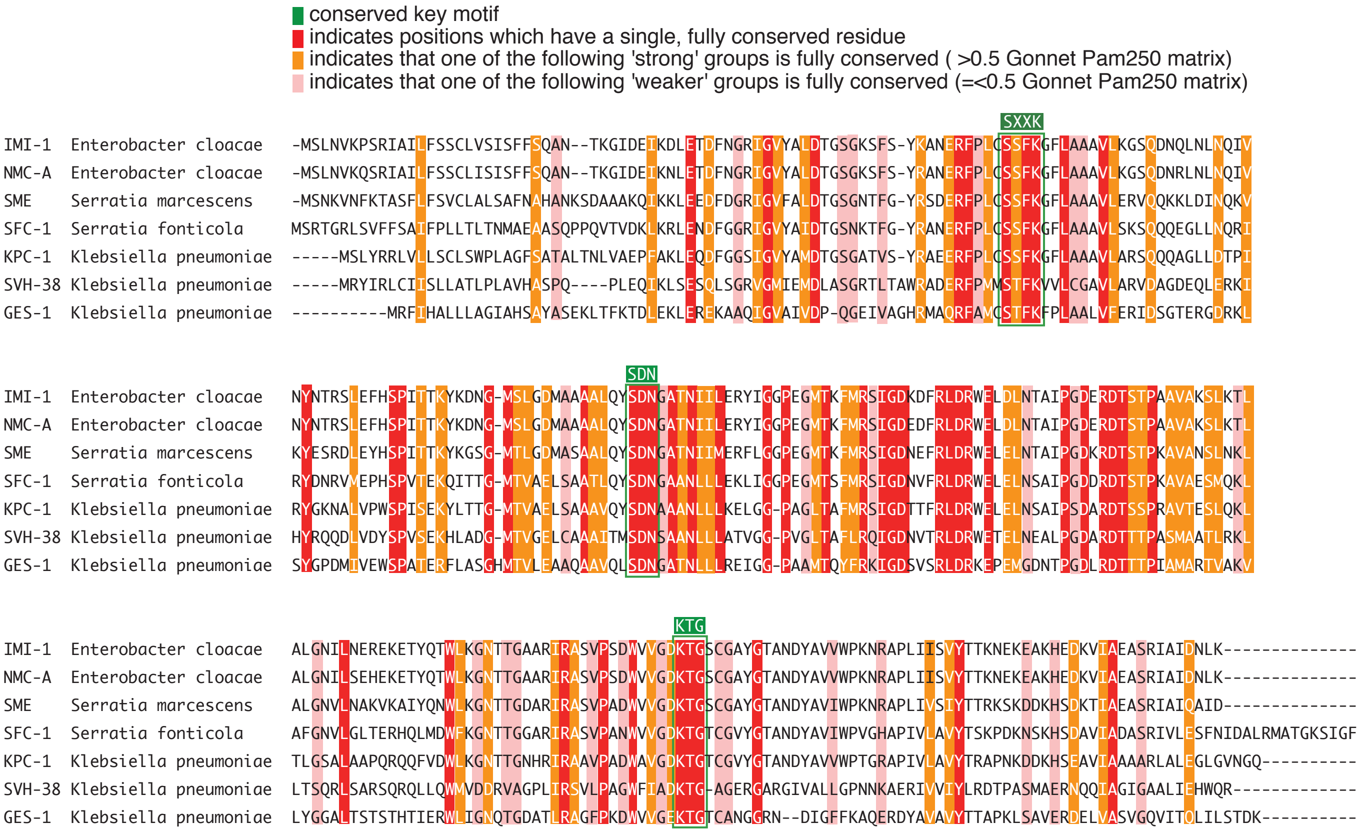

Supplemental Fig. 2. The alignment of class A carbapenemases based on their primary sequences

Characteristic features of class A β-lactamases are the tetrad motif S-X-X-K, the S-D-N triad, and the K-T-G triad, which contain the serine in the enzyme active site in their primary sequences. Based on the primary sequences, the tree was generated using Clustal Omega [32, 33, 34] of GenomeNet at Kyoto University Bioinformatics Center (https://www.genome.jp/tools-bin/clustalw).
